# Autonomic Modulation and Health-Related Quality of Life among Schizophrenic Patients Treated with Non-Intensive Case Management

**DOI:** 10.1371/journal.pone.0026378

**Published:** 2011-11-04

**Authors:** Li-Ren Chang, Yu-Hsuan Lin, Terry B. J. Kuo, Hung-Chieh Wu Chang, Chih-Min Liu, Chen-Chung Liu, Hai-Gwo Hwu, Cheryl C. H. Yang

**Affiliations:** 1 Department of Psychiatry, National Taiwan University Hospital, Yun-Lin Branch, Yunlin, Taiwan; 2 Department of Psychiatry, National Taiwan University Hospital, Taipei, Taiwan; 3 Institute of Brain Science, National Yang-Ming University, Taipei, Taiwan; 4 Sleep Research Center, National Yang-Ming University, Taipei, Taiwan; 5 Research Center for Adaptive Data Analysis, National Central University, Taoyuan, Taiwan; 6 Department of Psychiatry, College of Medicine, National Taiwan University, Taipei, Taiwan; Chiba University Center for Forensic Mental Health, Japan

## Abstract

**Background:**

Schizophrenia is associated with autonomic dysfunction and this may increase cardiovascular mortality. Past studies on autonomic modulation of schizophrenic patients focused on inpatients rather than individuals in a community setting, especially those receiving non-intensive case management (non-ICM). Besides, autonomic modulation and its association with health-related quality of life (HRQoL) in this population remain unexplored.

**Methods:**

A total of 25 schizophrenic patients treated by non-ICM and 40 healthy volunteers were matched by age, gender and body mass index; smokers were excluded. Between the two groups, we compared the individuals' 5 min resting assessments of heart rate variability and their HRQoL, which was measured using EuroQoL-5D (EQ-5D). Patients with schizophrenia were assessed for psychopathology using the Positive and Negative Syndrome Scale for Schizophrenia (PANSS). We examined the relationship between heart rate variability measurements, HRQoL scores, PANSS scores, and other clinical variables among the schizophrenic patients treated by non-ICM.

**Results:**

Compared to the controls, patients with schizophrenia showed a significant impairment of autonomic modulation and a worse HRQoL. Cardiovagal dysfunction among the schizophrenic patients could be predicted independently based on lower educational level and more negative symptoms. Sympathetic predominance was directly associated with anticholinergics use and EQ-5D using a visual analogue scale (EQ-VAS).

**Conclusion:**

Patients with schizophrenia treated by non-ICM show a significant impairment of their autonomic function and HRQoL compared to the controls. Since the sympathovagal dysfunction is associated with more negative symptoms or higher VAS score, the treatment of the negative symptoms as well as the monitoring of HRQoL might help to manage cardiovascular risk among these individuals. In addition, EQ-VAS scores must be interpreted more cautiously in such a population.

## Introduction

Schizophrenia is a chronic, debilitating mental disorder that affects approximately 1% of the general population [1]. It is associated with central autonomic system abnormalities and these may influence heart rate variability (HRV) [2], [3], baroreflex sensitivity [4], pupillary light reflex [5], and electrodermal activity [6]. These abnormalities may be associated with a disease-inherent genetic vulnerability [7]. The psychopathology of schizophrenia has been found to be associated with decreased vagal modulation [4], [5], [8]–[14] or overactive sympathetic function [6], [8], [12]. This autonomic dysfunction might be exacerbated by antipsychotic use [15], [16]. In addition, the lower functioning of patients with schizophrenia is associated with repressed overall autonomic and parasympathetic function [10].

Autonomic dysregulation may reduce the adaptability of the cardiovascular system to changes in the internal or external environment [15], [17]–[20], and thus increase the risk of life-threatening arrhythmias [21], myocardial infarction [22], heart failure [22], and sudden death [23]. Such effects seem to be associated with the cardiovascular mortality that is found among patients with schizophrenia [24], [25]. In a British community cohort of 370 patients with schizophrenia, an excess mortality was noted after 13-years of follow-up. The standardized mortality ratio (SMR) of patients with schizophrenia was significantly higher than in the general population, as were the SMRs for circulatory diseases and undetermined death [24]. The early identification of autonomic dysfunction in a community population might help with the provision of interventions that will minimize this mortality. However, most studies of autonomic modulation among patients with schizophrenia have focused on non-medicated [8], [10], [11], [17] or medicated subjects [10], [12], [15] in an inpatient setting. Such studies do not reflect the real-life condition of cardiac autonomic modulation among schizophrenic patients dwelling in community. The autonomic status of schizophrenic patients in a community setting might be different from that of patients in an inpatient setting because of their greater exposure to environmental risk factors [24].

Schizophrenic patients treated with non-intensive case management (non-ICM) constitute a significant community population of schizophrenics in Taiwan. According to the latest Cochrane review published in 2011 [26], non-ICM involves providing care at home or places of work with an assertive outreach, a multi-disciplinary team, and a caseload of over 20 people. Compared with ICM, with a caseload of less than 20 people, non-ICM is more feasible in developing countries due to its clinical effectiveness and lower cost. Non-ICM is usually indicated for schizophrenic patients who have poor drug compliance, frequent relapses or repeated hospitalization. Though this approach seems to be associated with less hospitalization, the autonomic modulation status of this population remains unexplored.

In addition to the above, the health-related Quality of life (HRQoL) of patients with schizophrenia is lower than that of the general population [27], [28]. HRQoL is defined as a patient's self-reported perception of his or her physical, emotional, mental, and functional wellbeing [29], and is a significant treatment outcome measure for patients with schizophrenia [30], [31]. Whether there is an association between HRQoL and HRV has not been explored in previous studies.

In this study, we compare time-domain and frequency-domain measures of HRV with assessments of the individual's HRQoL between schizophrenic patients treated by non-ICM and healthy controls. The two groups were matched by age, gender and body mass index (BMI); individuals using tobacco were excluded from this study. We further examined the relationship between HRV, HRQoL, psychopathology and other clinical variables in the schizophrenia group. We hypothesized that, firstly, the schizophrenia group would have worse HRV and lower HRQoL compared to the control group and, secondly, the worse HRV in schizophrenia group could be predicted by HRQoL and psychopathology.

## Materials and Methods

### Participants

This study was carried out by the Department of Psychiatry, National Taiwan University Hospital (NTUH), Yun-Lin Branch, in Taiwan. The Institutional Review Board of NTUH approved this study and all participants provided written informed consent. From Jan 2011 to May 2011, 25 schizophrenic patients were voluntarily recruited from the non-ICM program of our hospital, in which a total of 130 patients with severe mental illness were enrolled. This program is based on an assertive community treatment model [26] but there is a caseload of more than 20 patients. It includes: (1) two face-to-face sessions per month at home or at a place of work by a multi-disciplinary team including trained psychiatrists, psychiatric nurses, and social workers. Each session averages 30 minutes, (2) complete evaluation of symptoms, drug side effects and social functioning by the team members themselves, (3) an emphasis on medication compliance and persistent attempts to engage with uncooperative clients, i.e. “an assertive outreach”, (4) offering psychoeducation and counseling to the patient and their family members, (5) the provision of 24 hour emergency cover, (6) the allowance of patients and their families' calling the non-ICM team at any time during the week.

40 healthy volunteers were recruited from same local community by poster and internet advertisement during the index period. All clinical data was obtained by a board-certified psychiatrist. For this study, we only included participants who met the following criteria: (a) a DSM-IV diagnosis of schizophrenia, or, for the healthy controls, the absence of DSM-IV disorders; (b) the absence of comorbid major depressive or manic episodes, mental retardation, any organic brain syndrome, hypertension, diabetes mellitus, or other major systemic disease; (c) non-use of tobacco, alcohol or any illicit substance; (d) the absence of tricyclic antidepressants, β-blockers, chlorpromazine, or non-psychotropic medications that are documented to influence heart rate variability.

Age, sex and BMI were matched between the two groups. Thereafter, all participants were investigated by carrying out a 5-minute electrocardiogram (ECG) in the morning (09:00–12:00). They were instructed to avoid all types of food containing caffeine or alcohol on the morning of ECG recording. They were then evaluated using the EuroQoL-5D (EQ-5D) for HRQoL. Patients with schizophrenia were assessed for psychopathology using the Positive and Negative Syndrome Scale for Schizophrenia (PANSS), and the Global Assessment of Functioning (GAF) for functioning.

### Outcome measures

#### Heart rate variability

The procedure for HRV analysis was based on the standard method [32] and has been reported previously [33]. In brief, a lead I ECG was taken for 5 minutes in the daytime while each subject sat quietly and breathed normally. ECG signal acquisition, storage and processing were performed using a HRV analyzer (SS1C, Enjoy Research Inc., Taiwan). Signals were recorded using an 8-bit analog-to-digital converter with a sampling rate of 512 Hz. The digitized ECG signals were analyzed online, and were simultaneously stored on a hard disk for offline verification. The computer algorithm then identified each QRS complex and rejected each ventricular premature complex or noise according to likelihood using a standard QRS template. Normal and stationary R-R interval values (RR) were resampled and interpolated at a rate of 7.11 Hz to produce continuity in the time domain. This interpolation produced 2048 data points over 288 s, which was then used for the subsequent Fourier transformation.

Power spectral analysis was performed using fast Fourier transformation (FFT). The baseline shift was deleted, and a Hamming window was used to attenuate the leakage effect [34]. For each time segment (288 s, 2048 data points), our algorithm estimated the power spectrum density based on the FFT. The resulting power spectrum was corrected for attenuation resulting from the Hamming window. The power spectrum was subsequently quantified into the standard frequency-domain measurements as defined previously [32], [33], including variance (variance of RR-interval values), very low-frequency power (VLF, 0.003–0.04 Hz), LF (0.04–0.15 Hz), HF (0.15–0.40 Hz), total power (TP) and LF/HF, normalized LF (LF%). LF% was calculated from LF/(total power-VLF)×100. TP, VLF, LF, HF, and LF/HF were logarithmically transformed to correct for skewed distributions [33]. HF is considered to represent vagal control of the heart rate. Normalized LF (LF%) and the ratio LF/HF are considered by some investigators to reflect sympathetic modulations and to mirror the sympathovagal balance [32], [35], [36]. LF was initially interpreted as a marker of sympathetic modulation but has been found to reflect baroreflex function in more recent studies [37]. TP and LF are contributed to jointly by the sympathetic and parasympathetic nerves [32].

#### Positive and Negative Syndrome Scale (PANSS)

The severity of psychopathologic symptoms was evaluated with the Positive and Negative Syndrome Scale (PANSS) [38] by a board-certified psychiatrist. PANSS includes a positive symptoms subscale score, a negative symptoms subscale score, and a general psychopathology score. It is a semi-structured interview based on information relating to the previous week and includes 30 items and a 1–7 point continuum for each item. Higher scores reflect a higher severity of psychotic symptoms. Anxiety and depression scores, i.e. the 2^nd^ and 6^th^ item of general psychopathology, were singled out as independent variables due to their possibly individual effects on HRV [39], [40].

#### Global Assessment of Functioning (GAF)

The objective evaluation of the patients' clinical course and social functioning was based on Global Assessment of Functioning (GAF), which is a measure of an individual's overall psychological, social, and occupational functioning as rated by the clinician [1]. The range of the GAF score is between 0 and 100. A higher score means better functioning, while a lower score means poorer functioning.

#### EuroQoL-5D (EQ-5D)

The EQ–5D has five dimensions with three categories of severity; these dimensions are mobility, self-care, usual activities, pain/discomfort, and anxiety/depression (www.euroHRQoL.org). As there is, as yet, no available Chinese utility value, we adopted the most commonly used EQ–5D UK population value set. The EQ–5D UK time trade-off index (TTO) [41] ranges between 1 (full health) and −0.59 (0 is death). A TTO index is based on hypothetical trade-offs between length of life and symptoms. Only participants who fully completed the EQ–5D questionnaire were included. Although there is no unequivocally agreed threshold for a minimum clinically important change in the EQ–5D, thresholds of 0.07 points have been observed [42]. The visual analogue scale (VAS) records the respondent's self-rated health on a vertical, visual analogue scale where the endpoints are labeled with ‘Best imaginable health state’ (indicating 100 points) and ‘Worst imaginable health state’ (indicating 0 point). EQ-5D has been validated for assessing patients with schizophrenia [43]–[45] and the use of the Chinese version of EQ–5D has also been validated [46].

Other data collected with the outcome measures included demographic data (age, education, marital status, occupation), medical conditions (systolic and diastolic blood pressure, body mass index (BMI)), clinical variables (onset of illness, duration of non-ICM, hospitalization times), and current psychotropic medications (second generation antipsychotics (SGA) use, chlorpromazine equivalent dose of antipsychotics and the use of benzodiazepines, anticholinergics, mood stabilizers, and antidepressants).

### Statistical methods

All statistical tests were carried out using the SPSS version 15.0 for Windows (SPSS, Chicago, Ill). Descriptive statistics for the total sample were analyzed to categorize the patients in terms of clinical and psychosocial characteristics. The demographic data and blood pressure among the patient and healthy control groups were compared by Pearson's Chi-square (for categorical variables) and by independent t test (for continuous variables).

To test our first hypothesis, we used independent t test to compare EQ-5D values, the time-domain measure and frequency-domain measures of the HRV between the two groups. We used Pearson correlation coefficients to examine the associations between identifiably impaired measures of HRV, demographic data, medication use, psychopathology, functioning, and HRQoL. Subsequently, multivariate regression analysis with stepwise method was performed to examine the association between impaired measures of HRV and HRQoL. To control for the confounding factors, covariates which have significant correlation with HRV measures in univariate analysis would be included in the models. Model 1 contains EQ-VAS only. Other covariates were added stepwise in the final model. Statistical significance was assumed for *p*<0.05. Values are expressed as means ± standard deviation (SD).

## Results

### Demographic data and clinical variables

The demographic and psychopathological characteristics of the participants are summarized in Table 1. The age of the schizophrenia group ranged from 23 years to 55 years (mean age 41.3±9.2 years) and their illness duration varied from 6 years to 34 years (mean duration 19.6±7.9 years). As shown in Table 1, when matched by age, gender and BMI, patients with schizophrenia were less likely to be married or to be employed than members of the healthy group. They also had a lower educational level and had lower blood pressures. Their EQ-VAS scores were significantly lower than the control group (64.1±21.7 vs 79.9±11.7, p = 0.003).

**Table 1:** Sample Description.

	Schizophrenia group	Healthy control	Statistics		
	(N = 25)	(N = 40)	t (63)	*χ2*	p value
**Age, years**	41.3±9.2	41.2±12.7	−0.036		0.972
**Gender, N (%)**					
Male	10 (40)	19 (48)		0.350	0.554
Female	15 (60)	21 (52)			
**BMI, kg/m^2^**	25.0±4.8	24.7±2.9	−0.302		0.764
**Education (%)**					
College and above	2 (8)	14 (35)		18.766	<0.001
Senior high school	3 (12)	16 (40)			
Junior high school and below	20 (80)	10 (25)			
**Martial status(%)**					
Unmarried	15 (60)	13 (33)		14.669	<0.001
Married or cohabiting	4 (16)	25 (63)			
Divorced, separated, or widowed	6 (24)	2 (5)			
**Job (%)**		(N = 32)			
Employed	3 (12)	30 (94)		38.478	<0.001
Unemployed	22 (88)	2 (6)			
**Length of illness, years**	19.6 (±7.9)	—			
**Duration of non-ICM, months**	50.8 (±45.4)	—			
**Number of hospitalizations**	2.6 (±2.6)	—			
**Systolic blood pressure, mmHg**	117.0 (±13.1)	125.2 (±14.5)	2.296		0.025
**Diastolic blood pressure, mmHg**	73.6 (±9.2)	81.8 (±11.2)	3.072		0.003
**Medication, n (%)**					
Second-generation antipsychotics	18 (72)	—			
Benzodiazepines	13 (52)	—			
Anticholinergics	11 (44)	—			
Antidepressants	4 (16)	—			
Mood stabilizers	2 (8)	—			
**Chlorpromazine equivalent dose, mg**	242.0 (±227.2)	—			
**PANSS scores**					
Positive symptoms subscale	14.6 (±5.3)	—			
Negative symptoms subscale	17.8 (±7.0)	—			
General psychopathology	27.8 (±7.5)	—			
Total score	60.2 (±17.0)	—			
Anxiety (G2)	2.3 (±1.2)	—			
Depression (G6)	1.6 (±0.9)	—			
**CGI-Severity scale**	4.0 (±1.2)	—			
**GAF**	45.2 (±7.4)	—			
**EuroQol-5D**		(N = 32)			
EQ-TTO	0.85 (±0.18)	0.89 (±0.22)	0.637		0.527
EQ-VAS	64.1 (±21.7)	79.9 (±11.7)	3.495		0.003

Note: N refers to the number of cases. Values are means (±Standard Deviation).

PANSS, Positive and Negative Syndrome Scale; BMI, body mass index; GAF, global assessment of functioning; CGI, clinical global impression; EQ-TTO, EuroQoL-5D time-trade-off score; EQ-VAS, EuroQoL-5D score on visual analogue scale.

### Comparison of time-domain and frequency-domain measures between the schizophrenia group and the control group

As shown in Table 2, patients with schizophrenia had a shorter RR when compared to the controls. They also had smaller values for TP (6.83±1.19 vs 7.35±0.77, p = 0.033) and HF (4.46±1.57 vs 5.32±1.04, p = 0.010), which indicated parasympathetic dysfunction. In contrast, they had higher LF/HF than control (1.33±0.76 vs 0.94±0.69, p = 0.036), which reflected sympathetic predominance. However, there was no group difference for LF, which mirrors baroreflex function.

**Table 2:** Comparisons of heart rate variability measures between the schizophrenia group and the healthy control groups.

	Schizophrenia group	Healthy control	Statistics	
	(N = 25)	(N = 40)	t (63)	p value
**RR, ms**	718.07 (±97.60)	790.58 (±115.54)	2.608	0.011
**TP, ln (ms^2^)**	6.83 (±1.19)	7.35 (±0.77)	2.175	0.033
**HF, ln (ms^2^)**	4.46 (±1.57)	5.32 (±1.04)	2.661	0.010
**LF, ln (ms^2^)**	5.79 (±1.22)	6.26 (±0.81)	1.867	0.067
**LF/HF, ln (ratio)**	1.33 (±0.76)	0.94 (±0.69)	−2.137	0.036

Note: Values are means (±Standard Deviation).

Mean RR, mean of all RR intervals; TP, total power; HF, high-frequency spectral component; LF, low-frequency spectral component of HRV.

### Correlation analysis of the identifiably impaired measures of HRV in the schizophrenia group

In order to explore potential correlation with the impaired measures for HRV in the schizophrenia group, we conducted a correlation analysis. Table 3 shows the correlations between the impaired HRV measures (RR, TP, HF, LF/HF) and demographic data, medication use, psychopathology, functioning, and HRQoL. Mean RR was correlated with antidepressant use and the negative symptoms subscale score. Both TP and HF were correlated with educational level, the negative symptoms subscale score and EQ-VAS. TP was also correlated with age and length of illness. LF/HF was correlated with anticholinergics use and EQ-VAS. No correlations were detected between the impaired measures for HRV and depression, anxiety or functioning.

**Table 3:** Univariate correlation of heart rate variability measures in schizophrenia group (N = 25).

	RR, ms	TP, ln(ms^2^)	HF, ln(ms^2^)	LF/HF, ln(ratio)
**Age, year**	0.239	**−0.411** *	−0.380	0.123
**Gender (Female = 0, Male = 1)**	0.041	0.072	0.030	−0.050
**Educational level (below junior high school = 0, above = 1)**	0.217	**0.493** *	**0.542** **	−0.250
**Marital status (single, divorced, widowed = 0, married, cohabitate = 1)**	0.009	−0.216	−0.099	−0.259
**Job (no = 0, yes = 1)**	0.376	−0.026	<0.001	−0.011
**BMI, kg/m^2^**	0.356	−0.036	0.014	−0.107
**Length of illness, years**	−0.002	**−0.437** *	−0.369	0.118
**Duration of non-intensive case management, months**	−0.085	−0.225	−0.229	0.239
**Number of hospitalizations**	−0.100	0.014	−0.030	0.163
**Medication**				
Second generation antipsychotics (No = 0, Yes = 1)	−0.194	0.161	0.156	−0.038
Antipsychotic dose (chlorpromazine equivalent dose)	−0.305	−0.266	−0.278	−0.031
Benzodiazepines (No = 0, Yes = 1)	0.207	−0.073	−0.111	0.104
Anticholinergics (No = 0, Yes = 1)	0.216	0.037	−0.124	**0.411** *
Antidepressants (No = 0, Yes = 1)	**−0.406** *	−0.174	−0.327	0.050
Mood stabilizers (No = 0, Yes = 1)	0.239	0.213	0.139	−0.076
**PANSS scores**				
Positive symptoms subscale	−0.226	−0.182	−0.308	0.150
Negative symptoms subscale	**−0.474** *	**−0.433** *	**−0.404** *	0.184
General psychopathology	−0.207	−0.250	−0.220	−0.110
Total score	−0.357	−0.345	−0.359	0.074
Anxiety (G2)	−0.352	0.005	0.003	−0.054
Depression (G6)	−0.336	−0.259	−0.173	−0.214
**CGI-Severity scale**	−0.356	−0.018	−0.116	0.120
**GAF**	0.217	0.319	0.374	−0.203
**EuroQol-5D**				
EQ-TTO	0.143	0.299	0.306	−0.083
EQ-VAS	−0.173	**−0.440** *	**−0.524** **	**0.444** *

Note: RR, mean of all RR intervals; TP, total power; HF, high-frequency spectral component; LF, low-frequency spectral component of HRV.

*p<0.05.

**p<0.01.

***p<0.001.

PANSS, Positive and Negative Syndrome Scale; BMI, body mass index; GAF, global assessment of functioning; CGI, clinical global impression; EQ-TTO, EuroQoL-5D time-trade-off score; EQ-VAS, EuroQoL-5D score on visual analogue scale.

### Multivariate stepwise regression analysis of the identified impaired measures for HRV in schizophrenia group

The determinants of each impaired HRV parameter of schizophrenic patients treated with non-ICM are shown in table 4. In model 1, which included HRQoL as the only covariate, EQ-VAS was a significant negative determinant of TP, HF and a positive determinant for LF/HF. In the final model, EQ-VAS remained positively associated with only LF/HF (*β* = 0.430, *p* = 0.023) after other covariates were adjusted for. In comparison, the effect of EQ-VAS on TP and HF became insignificant after adding other covariates into the regression model. Notably, a borderline statistical significance was noted in the association between EQ-VAS and HF (*β* = −0.349, *p* = 0.057). TP and HF were predicted by educational level (*β* = 0.459, 0.509, respectively) and negative symptoms subscale score of PANSS (*β* = −0.393, −0.356, respectively) independently. Anticholinergics use remained positively associated with LF/HF after controlling for other covariates. The models predicting RR, TP, HF, and LF/HF were statistically significant with *R^2^* of 0.263, 0.399, 0.419, and 0.359. There was no significant effect of age, length of illness, or antidepressants use on HRV parameters in regression models.

**Table 4:** Multiple linear regression models predicting heart rate variability measures in schizophrenia group (N = 25).

Predictors	RR, ms	TP, ln (ms^2^)	HF, ln (ms^2^)	LF/HF, ln (ratio)
**Model 1:** *R^2^*	0.030	**0.194** *	**0.274** **	**0.197** *
	*β (B±S.E.)*			
EQ-VAS	−0.173	**−0.440** * **(−0.025±0.011)**	**−0.524** ** **(−0.039±0.013)**	**0.444** * **(0.016±0.007)**
**Final Model:** *R^2^*	**0.263** *	**0.399** **	**0.419** **	**0.359** **
	*β (B±S.E.)*			
EQ-VAS	−0.148	−0.270	−0.349	**0.430** * **(0.015±0.006)**
Age	0.173	−0.336	−0.262	−0.145
Educational level	0.197	**0.459** * **(1.340±0.496)**	**0.509** ** **(1.967±0.646)**	0.042
Length of illness	0.063	−0.218	−0.102	−0.138
PANSS-Negative symptoms subscale	**−0.513** * **(−7.048±2.515)**	**−0.393** * **(−0.067±0.029)**	**−0.356** * **(−0.080±0.038)**	0.196
Anticholinergics	0.138	0.118	−0.036	**0.403** * **(0.623±0.270)**
Antidepressants	−0.283	0.037	−0.137	0.028

Note: RR, mean of all RR intervals; TP, total power; HF, high-frequency spectral component; LF, low-frequency spectral component of HRV.

*p<0.05.

**p<0.01.

***p<0.001.

B: unstandardized coefficient; β: standardized coefficient; S.E.: standard error; PANSS, Positive and Negative Syndrome Scale; EQ-VAS, EuroQoL-5D score on visual analogue scale.

## Discussion

Although autonomic dysfunction in patients with schizophrenia has been validated in previous studies, it is much less explored in patients in community settings, especially those treated by non-ICM. We also do not know the extent of and the correlations with autonomic dysfunction in this population. Our study is pilot one in the field of community psychiatry because we have studied exclusively autonomic dysfunction and its determinants among schizophrenic patients who are being treated by non-ICM. We try to investigate the “real-world” condition of autonomic modulation among patients dwelling in community, rather than in laboratories. Furthermore, our patient population is special because the duration of illness is among the longest in HRV studies of schizophrenia (averagely 19.6 years in our study, versus less than 10 years in most studies) [11]–[13]. Our result is also pioneering in exploring the relationship between HRV and HRQoL in patients with schizophrenia. We have carefully controlled for age, gender, and BMI, and excluded tobacco use, alcohol use, illicit drug use, major systemic disease and other psychiatric illnesses for the two populations. Our findings support the first hypothesis, namely that schizophrenic patients treated with non-ICM show a significant impairment of cardiac autonomic modulation and a lower HRQoL compared to the control individuals. They have a lower mean RR, an impaired HRV, a repressed parasympathetic function, and a sympathetic predominance. Our findings only partially support our second hypothesis. In this context, we were able to show that the reduced vagal function was associated with severer psychopathology of negative symptoms; HRQoL, as indicated by EQ-VAS, was associated with sympathetic predominance.

As expected, total autonomic activity, parasympathetic function and sympathovagal balance, as indicated by TP, HF and LF/HF, were found to be worse in the schizophrenia group compared to the control group. This supports previous findings [4]–[6], [8]–[12]. These patterns of autonomic dysfunction persists even when patients with schizophrenia are treated with antipsychotics [10], [15], [16]. Our results are also consistent with this finding. Williams and colleagues proposed that patients with paranoid schizophrenia have impairment of their higher centers within the central autonomic network (CAN) in addition to the presence of peripheral autonomic dysfunction. Lack of activation in the medial prefrontal cortex compromises inhibitory control over amygdale-driven autonomic function [47]. The subsequent autonomic dysfunction possibly causes recurrent arrhythmia or sudden cardiac death in patients with schizophrenia [11], [48], [49]. Monitoring such cardiovascular dysfunction might be beneficial to patients with schizophrenia and help to reduce cardiovascular mortality.

Our results indicated the association between vagal activity and negative symptoms. Impaired vagal function has previously been found to be related to more pronounced positive symptoms [11], [13], [19] or to the total score of PANSS [14], [19], [20]. However, we found the correlation between TP, HF and negative symptom subscale score. This correlation remained significant after controlling for potential confounders in multiple regression models. The relationship between HRV and negative symptoms has been much less reported [13]. In a population of acutely ill schizophrenic patients, Boettger and colleagues [13] found the correlation between HRV and positive symptoms. They also noticed a significant correlation between negative symptoms and PD^mHF^ (mean high frequency peak decay), a novel non-linear measure representing vagal information flow. The correlation was positive during the day (r = 0.457), which was consistent with our result since higher PD^mHF^ values represented lower vagal information flow. Interestingly, this correlation turned to be negative during the night (r = −0.467), which reflected altered diurnal autonomic variation [13]. Negative symptoms include affective flattening, poverty of thoughts, and avolition [1]. They may be affected by CAN, which regulates affective, cognitive and motivational processes in the brain [2], [3].Whether this exclusive association between negative symptom and vagal activity in our patients could be explained by their chronic course of disease (up to 20 years averagely) remained speculative. We still have very little understanding of this relationship as well as diurnal regulation between negative symptom and vagal function. Its neurophysiological mechanism needs further investigation.

In univariate analysis, we found that TP, HF, and LF/HF were all correlated with EQ-VAS. After controlling for demographic data, psychopathology and medication, only the association between LF/HF and EQ-VAS remained. The statistical significance of the relationship between TP, HF, and EQ-VAS became marginal. The relationship between impaired cardiac autonomic function and HRQoL in patients with schizophrenia has not been investigated. In a normal population, self-reported quality of life and depression might affect HRV [50]. Among patients with chronic obstructive pulmonary disease, HRV is directly linked to the perceived quality of life [51]. However, the finding that schizophrenic patients with more sympathetic predominance report better well-being went beyond our expectation. The overestimation of HRQoL by schizophrenic patients might partially explain this phenomenon. While the EQ-TTO score is estimated from five dimensions (mobility, self-care, usual activities, pain or discomfort, and anxiety or depression), the EQ-VAS score is rated by participants intuitively. The latter assesses well-being in a highly subjective manner. It is possible that patients with schizophrenia might have an unrealistic appreciation of self and the outer world due to cognitive deficits as well as reasoning bias [52]. Recently, Beck and colleagues [53] found that schizophrenic patients with deficit syndrome had more defeatist attitudes but higher self-esteem than those without deficit syndrome. These attitudes might lead to social withdrawal and protect the self-esteem. Thus they might report an unrealistically high quality of life to the community mental health team though they had poor mental or physical condition actually. Sympathetic predominance *per se*, or associated vagal withdrawal, may increase cardiovascular risk [54], [55]. In these circumstances, the potential cardiovascular risk might be overlooked. The neurophysiological basis for this association remains unclear. While prefrontal hypoactivity is related to cognitive deficits in schizophrenia, it may fail to inhibit subcortical sympathoexcitatory circuits and lead to subsequent sympathetic predominance [55]. Future research should direct toward exploring the roles of dysfunctional attitudes and neurocognition in the relationship between HRV and HRQoL. Clinicians must be aware that patients may report feeling well, but they may, nevertheless, have autonomic dysfunction. Careful monitoring of autonomic status is therefore important among schizophrenic patients living in community and subjective quality of life rating measures, especially EQ-VAS, should be interpreted with caution among this population.

Our study indicated positive association between educational level and vagal function, which supported previous findings [56]. Patients with higher educational level may lead healthier lifestyle and have better cognitive capacities, which are related to better HRV [56], [57]. It might be helpful to monitor autonomic function of patients with lower educational level and to provide them relevant psychoeducation. Besides, we noticed the positive association between anticholinergics use and sympathetic predominance. Anticholinergics were mostly reported to reduce parasympathetic function in previous research [58]. In our patients, the autonomic imbalance in the sympathetic direction was also accompanied by vagal withdrawal. The use of anticholinergics should be cautious in patients with schizophrenia since it is associated with sympathovagal imbalance. Although no correlation between cardiovagal function and antipsychotic use was identified, antipsychotic use may still exacerbate autonomic dysfunction [15], [16]. The impact of long-term antipsychotic use on autonomic modulation of chronic patients as well as its relationship with cardiac toxicity deserve further investigation [59]. Our results showed no association between the patient's functioning and lower measures of HRV, which were different from Fujibayashi et al. 's findings [10]. The measure of GAF is simplified, and more comprehensive evaluation of functionality is needed to clarify the inconsistent results.

There are several limitations to this study. Firstly, we did not include evaluations of neuro-cognitive functions, drug compliance, and any side effects of the patient's medication. Therefore, there is the possibility that these factors might influence HRV. Some disease-independent factors, such as physical fitness and unhealthy lifestyle, may also influence HRV and are hard to control for [13], [60]. For example, aerobic exercises can increase parasympathetic tone and decrease sympathetic activity [60]. Secondly, the sample size is limited due to the need to carefully match for age, gender, and educational level and to exclude tobacco use and comorbid conditions. In future research, larger sample size is needed to validate the preliminary findings of this study. Thirdly, there were four patients (16%) in our study population taking antidepressants (two used fluoxetine, two sertraline). Evidences regarding whether antidepressants deteriorate HRV or not remains inconclusive, as are seen in most recent large-scale studies and their consequent debates [39], [61]–[63]. However, Koschke and colleagues [64] suggested differential autonomic effects of serotonin and noradrenaline selective reuptake inhibitor (SNRI) as well as selective serotonin reuptake inhibitor (SSRI) by novel non-linear techniques. Though we did not find significant associations between HRV and antidepressant use in our current sample (Table 4), our results should be interpreted cautiously. Fourthly, we were unable to evaluate possible discriminative influences on autonomic function by the various antipsychotic agents because SGA use and the chlorpromazine equivalent dose do not represent autonomic effects of different agents. Fifthly, though the 5-minutes measure of HRV revealed detailed information about autonomic modulation of RR intervals [32], it did not have advantages owned by 24-hours ECG recordings including less mathematical errors, less influenced by fluctuations of autonomic functions, as well as assessment of diurnal changes [13]. This is a limitation of our study. Sixthly, the results of a non-ICM program should not be generalized to patients in other community settings or institutions due to differences in the clinical characteristics of the patients. Up to the present there have been no trials that compare the clinical characteristics of non-ICM patients with standard care patients [26]. Thus the lack of a comparison group receiving standard care precludes direct investigation of non-ICM characteristics. Finally, our study was cross-sectional in nature, which means that it is not possible to generate any conclusions regarding causality between the variables.

As an important treatment model of community mental health, non-ICM components are as pragmatically necessary as hospital services in all areas regardless of the level of resources available [65]. Despite clinical effectiveness, non-ICM is still associated with significant impairment in HRV and HRQoL when compared with a healthy population. Since sympathovagal imbalance is associated with psychopathology of negative symptoms and higher VAS score, the treatment of negative symptoms as well as the monitoring of HRQoL might help to manage cardiovascular risk among these individuals. The subjective rating of EQ-VAS score should be interpreted cautiously in schizophrenic patients treated with non-ICM.
